# The *GhmiR157a–GhSPL10* regulatory module controls initial cellular dedifferentiation and callus proliferation in cotton by modulating ethylene-mediated flavonoid biosynthesis

**DOI:** 10.1093/jxb/erx475

**Published:** 2017-12-14

**Authors:** Lichen Wang, Nian Liu, Tianyi Wang, Jianying Li, Tianwang Wen, Xiyan Yang, Keith Lindsey, Xianlong Zhang

**Affiliations:** 1 National Key Laboratory of Crop Genetic Improvement, Huazhong Agricultural University, Wuhan, Hubei, P. R. China; 2 Department of Biosciences, Durham University, South Road, Durham, UK

**Keywords:** Callus proliferation, cotton, ethylene, flavonoids, *GhmiR157a–GhSPL10*, somatic embryogenesis

## Abstract

MicroRNAs (miRNAs) modulate many biological processes through inactivation of specific mRNA targets such as those encoding transcription factors. A delicate spatial/temporal balance between specific miRNAs and their targets is central to achieving the appropriate biological outcomes. Somatic embryogenesis in cotton (*Gossypium hirsutum*), which goes through initial cellular dedifferentiation, callus proliferation, and somatic embryo development, is of great importance for both fundamental research and biotechnological applications. In this study, we characterize the function of the *GhmiR157a*–*GhSPL10* miRNA–transcription factor module during somatic embryogenesis in cotton. We show that overexpression of *GhSPL10*, a target of *GhmiR157a*, increases free auxin and ethylene content and expression of associated signaling pathways, activates the flavonoid biosynthesis pathway, and promotes initial cellular dedifferentiation and callus proliferation. Inhibition of expression of the flavonoid synthesis gene *F3H* in *GhSPL10* overexpression lines (*35S:rSPL10-7*) blocked callus initiation, while exogenous application of several types of flavonol promoted callus proliferation, associated with cell cycle-related gene expression. Inhibition of ethylene synthesis by aminoethoxyvinylglycine treatment in the *35S:rSPL10-7* line severely inhibited callus initiation, while activation of ethylene signaling through 1-aminocyclopropane 1-carboxylic acid treatment, *EIN2* overexpression, or inhibition of the ethylene negative regulator CTR1 by RNA interference promoted flavonoid-related gene expression and flavonol accumulation. These results show that an up-regulation of ethylene signaling and the activation of flavonoid biosynthesis in *GhSPL10* overexpression lines were associated with initial cellular dedifferentiation and callus proliferation. Our results demonstrate the importance of a *GhmiR157a*–*GhSPL10* gene module in regulating somatic embryogenesis via hormonal and flavonoid pathways.

## Teaser

Ethylene-mediated flavonoid biosynthesis is controlled by a *GhmiR157*–*GhSPL10* regulatory module necessary for initial cellular dedifferentiation and callus proliferation during cotton somatic embryogenesis.

## Introduction

Plant cells exhibit developmental plasticity manifested by diverse tissue or organ regeneration pathways, which can be classified into two main types, *de novo* organogenesis and somatic embryogenesis (SE) (Ikeuchi *et al.*, 2016).

Ethylene plays an important hormonal role during plant growth and development. The ethylene signaling pathway is initiated from a receptor complex that includes ETHYLENE RESISTANT1 (ETR1), which in the presence of ethylene inactivates the negative regulator CONSTITUTIVE TRIPLE RESPONSE1 (CTR1), leading to ethylene responses via ETHYLENE INSENSITIVE2 (EIN2), EIN3 and ETHYLENE RESPONSE FACTOR1 (ERF1), with the assistance of the F-box proteins EBF1 and EBF2 (Yu and Huang, 2017). Several pieces of evidence implicate ethylene as an endogenous stimulator of cell division. For example, ethylene can stimulate root stem cell division (Ortega-Martínez *et al.*, 2007) and reunion of disconnected stem tissue by stimulating pith cell division in Arabidopsis (Asahina *et al.*, 2011). Ethylene also promotes cambium cell division in *Populus* during tension wood production (Love *et al.*, 2009).

Ethylene has been found to induce callus formation and plant regeneration in several species. Ethylene promoted callus formation in citrus bud explants in culture (Goren *et al*. 1979). During apple callus proliferation, endogenous ethylene concentrations rose rapidly before declining (Lieberman *et al.*, 1979). In barley, endogenous ethylene content was closely related to regeneration competency, and application of the ethylene precursor 1-aminocyclopropane 1-carboxylic acid (ACC) promoted regeneration (Jha *et al.*, 2007). In Arabidopsis, inhibition of ethylene responses by Ag_2_S_2_SO_3_ in wild type and ethylene-insensitive mutants *etr1-1*, *ein2-1*, *ein4*, and *ein7* reduced the rate of shoot regeneration, while the constitutive ethylene response mutants *ctr1-1* and *ctr1-12* and the ethylene overproduction mutant *eto1* showed increased shoot regeneration (Chatfield and Raizada, 2008). Ethylene improved SE in soybean, whereby overexpression of *GmAGL15* promoted embryogenesis by activating expression of *ACS*, *ACO* and *ERF*; and in wild type, 25 µM ACC treatment promoted SE, while 10 µM aminoethoxyvinylglycine (AVG), 100 µM CoCl_2_, or 10 µM Ag_2_S_2_SO_3_ significantly inhibited SE (Zheng *et al.*, 2013*a*). Endogenous ethylene content has been found to be positively related to SE competence in soybean (Zheng *et al.*, 2013*b*).

Flavonoid biosynthesis is a branched secondary metabolic pathway of phenylpropanoid metabolism, and involves the key enzymes chalcone synthase (CHS), chalcone isomerase (CHI), flavanone 3-hydroxylase (F3H), flavonoid 3′-hydroxylase (F3′H), and flavonol synthase (FLS) for flavonol biosynthesis, and dihydroflavonol 4-reductase (DFR), anthocyanidin synthase, and anthocyanidin reductase for anthocyanin biosynthesis (Liu *et al.*, 2015). The flavonoid biosynthesis pathway was up-regulated in a *Medicago truncatula* line exhibiting a 500-fold increased capacity to regenerate plants by SE compared with a parent line (Imin *et al.*, 2008), suggesting that flavonoids might have a positive effect on plant regeneration. Recently, polyphenol and flavonoid accumulation was demonstrated during callus proliferation in *Psyllium*, implying that the antioxidant activity of flavonoids might contribute to callus induction and SE (Talukder *et al.*, 2016). Evidence has also been found for the involvement of flavonoids in somatic embryo maturation in cacao (Maximova *et al.*, 2014).

Ethylene and auxin are reported to induce flavonol accumulation through MYB12, which is a positive regulator of flavonol biosynthetic enzyme genes (Lewis *et al.*, 2011). Expression levels of *CHS* and *FLS* were down-regulated in the ethylene-insensitive *ein2* mutant. The relatively high expression of ethylene- and flavonoid-related genes during somatic embryo maturation in cacao also links ethylene with flavonoid biosynthesis and SE (Maximova *et al.*, 2014).

Auxin homeostasis is also crucial for SE in both Arabidopsis and cotton. In Arabidopsis the transcription factor genes *LEAFY COTYLEDON1* (*LEC1*) and *LEC2* are necessary and sufficient for SE, as ectopic expression of either transcription factor promotes SE and *lec* mutants have defects in SE (Lotan *et al.*, 1998; Stone *et al.*, 2001; Gaj *et al.*, 2005). Activation of the auxin biosynthesis-related *YUC* genes by LEC2 may regulate auxin supply during SE (Stone *et al.*, 2008). In cotton, endogenous auxin is also elevated in embryonic callus compared with non-embryonic callus (Yang *et al.*, 2012). Disruption of auxin homoestasis by overexpression of *GhCK1*, which acts downstream of *GhLEC1*, resulted in defective embryogenesis (Min *et al.*, 2015).

MicroRNAs (miRNAs) are ~20–25 nt non-coding RNAs that are essential components of the gene silencing machinery in most eukaryotic organisms and direct the post-transcriptional silencing of target mRNA or transcriptional silencing through DNA methylation during development (Rogers and Chen, 2013; Achkar *et al.*, 2016). For example, miR156-targeted *SQUAMOSA-PROMOTER BINDING PROTEIN-LIKE* (*SPL*) transcription factor RNAs have been demonstrated as key regulators participating in multiple biological processes in plants, such as reproductive phase change, leaf development, tillering and branching, panicle or tassel architecture, fertility and fruit ripening, and responses to biotic and antibiotic stresses (Wang and Wang, 2015). Moreover, miR156-regulated *SPL* RNAs accumulate with ageing and result in the gradual reduction in shoot regenerative capacity through genetic interaction with the B-type ARABIDOPSIS RESPONSE REGULATORs, and attenuate the cytokinin signaling pathway (Zhang *et al.*, 2015).

The research reported here aims to understand the biological events modulated by *GhmiR157a–GhSPL10* interaction during SE in cotton. We show that *GhmiR157a* and its target *GhSPL10* had opposite expression patterns during the embryonic transition stage in the process of SE in cotton, and overexpression of *GhSPL10* promotes callus proliferation by activating hormone signaling pathways, particularly ethylene-mediated flavonol biosynthesis.

## Materials and methods

### Plant material and growth conditions

*G. hirsutum* cv. YZ1 was used as wild type, and transgenic plants were all in the YZ1 background. Seeds of wild type and transgenic plants were germinated for 5 d on 1/2 Murashige and Skoog (MS) medium under dark, as described previously (Yang *et al.*, 2012). Hypocotyls of etiolated seedlings were sampled at 0 h, or cut into 5–7 mm sections as explants to induce callus on MSB medium for different time points or subculture for embryogenic callus and somatic embryos as described previously (Yang *et al.*, 2012). Five-day-old etiolated seedlings were photographed and hypocotyl lengths were measured.

### Plasmid construction and genetic transformation

A *GhmiR157a* overexpression vector was generated previously (Liu *et al.*, 2017). The full-length coding sequence (stop codon removed, 1059 bp) together with a 29 bp upstream fragment of *GhSPL10* (*Gh_A12G0866*) was cloned from cotton embryo cDNA and ligated to pGEM-T easy vector. From this the *GhmiR157a*-resistant *GhSPL10* (*rSPL10*) was generated by introducing seven mutations into the predicted *GhmiR157a* binding site using recombinant PCR. It was cloned into a Gateway entry vector, and subsequently recombined into a C-terminal 6xhistidine fusion vector, pGWB408, which harbors a 35S promoter using Gateway LR clonase II enzyme mix (Invitrogen), for overexpression studies. The full-length coding sequence of *EIN2* was cloned from Arabidopsis (*AT5G03280*) and ligated to pCAMBIUM2300 to generate an overexpression vector. The RNA interference (RNAi) region of *GhCTR1* (coding sequence of Gh_D09G1340 from 2162 to 2556 bp, plus a 83 bp fragment of the 3′-untranslated region) was cloned into the RNAi vector pHellsgate4 by recombination. The primers for vector construction described above are listed in Supplementary Table S1 at *JXB* online. All overexpression and RNAi vectors were introduced into cotton (YZ1) plants by *Agrobacterium tumefaciens* (strain EHA105)-mediated transformation as previously described (Jin *et al.*, 2006). Segregating wild type plants harboring no transgenes from self-pollinated *35S:rSPL10-7* overexpression line hemizygotes were used as null controls. A *F3H* RNAi line was generated and termed *F3H-Ri3* as previously described (Tan *et al.*, 2013).

### ACC, AVG, CoCl_2_, Ag_2_S_2_SO_3_, indole-3-acetic acid, and flavonoid treatments during callus induction

For ethylene-related treatments during *in vitro* culture, a final concentration of 10 μM ACC, 5 μM AVG, 100 μM CoCl_2_, or 10 μM Ag_2_S_2_SO_3_ was added to the MSB medium, and hypocotyl explants of etiolated seedlings grown under standard conditions were cultured for 2 weeks. Callus morphology was monitored and callus proliferation rate (CPR; see below) was calculated. At 3, 6, and 9 h, flavonoid biosynthesis-related gene expression was analysed, and at 1, 3, and 5 d, flavonols were quantified. For the *F3H* qRT-PCR assay, 25 μM ACC was added to the MSB medium and explants were cultured for 9 h before analysis.

For auxin treatment during *in vitro* culture, 1 μM indole-3-acetic acid (IAA) was added to the MSB medium, on which hypocotyl explants of normally grown etiolated seedlings were cultured for 2, 4, and 6 d for flavonoid-related gene expression analysis. For flavonoid treatments during *in vitro* culture, 10 μM dihydroquercetin (DHQ), kaempferol (K), or quercetin (Q) was added to the MSB medium, on which the hypocotyl explants from etiolated seedlings grown under standard conditions were cultured for 1, 2, and 3 weeks for callus morphological observation and CPR calculation, or for 2, 4, and 6 days for cell cycle-related gene expression analysis. MSB medium supplemented with 2.5, 5, or 10 μM Q, or 2.5 μM dihydrokaempferol (DHK), DHQ, K, and Q (abbreviated as DDKQ), was used for explant culture and CPR calculation post 2 weeks.

All chemicals were filter sterilized, and medium with no supplemental chemicals was defined as mock.

### Southern blotting, qRT-PCR, RT-PCR, and RNA ligase-mediated rapid amplification of 5′-cDNA ends

Total DNA was extracted using the Plant Genomic DNA Kit (Tiangen, China). Southern blotting was performed following enzyme digestion of genomic DNA, electrophoresis and hybridization. The PCR fragments of *NPTII* were used as a probe. The detailed methods were reported previously (Li *et al.*, 2010).

Total RNA was extracted using a modified guanidine thiocyanate method (Zhu *et al.*, 2005) or the RNAprep Pure Plant Kit (Tiangen, China). Reverse transcription of RNA for miRNA qRT-PCR was performed using stem–loop primers as described (Varkonyi-Gasic *et al.*, 2007). qRT-PCR and RT-PCR were performed as described previously (Jin *et al.*, 2014). Relative expression levels of genes were determined by 2^−Δ*C*t^ with *UBQ7* as endogenous reference (Tu *et al.*, 2007). Rapid amplification of 5′-cDNA ends (5′-RACE) of *GhSPL10* was performed using a GeneRacer kit (Invitrogen). Briefly, RNA fragments were ligated to RNA adapters and transcribed using the GeneRacer Oligo(dT) primer. Nested PCR amplifications were performed with 5′ adaptor primers and 3′ gene-specific primers according to the manufacturer’s instructions. Finally, the PCR products were cloned to the pGEM-T easy vector, and 12 positive *E. coli* clones were sequenced. All primers used are listed in Supplementary Table S1.

### Callus proliferation rate calculation and histocytological analysis

CPR was calculated as the fold change of the weight gained by explants, i.e. change in weight per unit time divided by the initial weight of the explants.

Excised hypocotyls were cultured on MSB medium for 5 d, and somatic embryos were fixed in 50% FAA [10% formalin, 5% acetic acid, and 50% ethanol (v/v)], dehydrated, cleared, and embedded as described previously (Jackson, 1991). Histological sections of 10 µm thickness were stained with toluidine blue (0.05%) and mounted in DPX resin (Sigma-Aldrich). For whole mount *in situ* hybridization, a locked nucleic acid probe with both ends with the digoxigenin modification was used as a negative control (scramble-miR, 5′-GTGTAACACGTCTATACGCCCA-3′, Exiqon), and 10 pmol probe was used for each slide. Probes for *GhSPL10* were generated by amplifying the 403 bp upstream, the 372 bp middle, and the 436 bp downstream fragments calculated from the start codon of *GhSPL10* transcript, ligated to pGEM-T vector (Promega) for sequencing to select the plasmids possessing the target sequences in sense orientation. Linearized plasmids from the end of T7 promoter were used as templates for the antisense probe synthesis using SP6 polymerase (Roche). Then the probes were hydrolysed in 200 mM carbonate buffer (pH 10.2) to be about 150 bp and mixed together for hybridization. Whole mount *in situ* hybridization was performed as described (Rosen and Beddington, 1993). Hybridization and washing steps were performed at 55 °C. Sections were observed by light microscopy. Primers used for probe amplification are listed in Supplementary Table S1.

### RNA sequencing and data analysis

Total RNA was isolated from hypocotyls of 5-day-old seedlings of null and *35S:rSPL10-7* lines growing under dark on 1/2 MS medium, at ~29 °C using a modified guanidine thiocyanate method (Zhu *et al.*, 2005), using two biological replicates. RNA libraries were constructed and sequenced with an Illumina HiSeq^TM^ 2000 at the Beijing Genomics Institute (BGI, Shenzhen, China). High throughput data processing and determination of differentially expressed genes was as described (Liu *et al.*, 2017).

### Quantification of endogenous IAA and ethylene content

For measurement of IAA content, ~0.2 g hypocotyl sections of 4-day-old etiolated seedlings were ground in liquid nitrogen and homogenized in 750 μl of 80% (v/v) methanol containing 10 ng ml^−1^ [^2^H_5_]IAA (OIChemlm Ltd, CAS: 76937-78-5) as internal standard and then shaken at 4 °C overnight. The supernatant was collected and another 450 μl of 80% methanol was added to the pellet for another 3 h shaking. The supernatant was twice extracted, mixed, and evaporated, and redissolved in 300 μl 50% (v/v) methanol and then filtered through nylon membranes with 0.22 μm aperture. The quantification of endogenous IAA was performed as described previously (Liu *et al.*, 2012).

For ethylene quantification, about five seeds germinated for 24 h in the dark on 1/2 MS medium were collected and sealed in a 12 ml vial with an air-tight cap at room temperature in the dark for 12 h; subsequently 1 ml gas was sampled from the vial by an air-tight syringe (Agilent) and injected into a gas chromatograph (7890A-5975C, Agilent). The standard curve was created with standard concentrations of ethylene: 1, 2, 3, and 5 ul/L, with the equation *y*=12.51*x−*0.914; *r*^2^=0.998.

### Flavonol measurements

Around 0.2 g hypocotyls or explants of tested lines, cultured for specific time periods, were ground in liquid nitrogen, and flavonols were extracted in 1 ml of 80% methanol and shaken at 4 °C overnight. The supernatant was evaporated and redissolved in 300 μl 50% methanol, which was further filtered through a 0.22 μm nylon membrane. The quantification of flavonols was as previously described (Tan *et al.*, 2013).

### Statistical analysis

All statistical analysis was based on at least two biological and three technical replicates, and the significance was determined by multiple comparisons using Statistix software (version 8.0).

## Results

### *GhSPL10* phylogeny and expression pattern during cotton SE

**Fig. 1.**
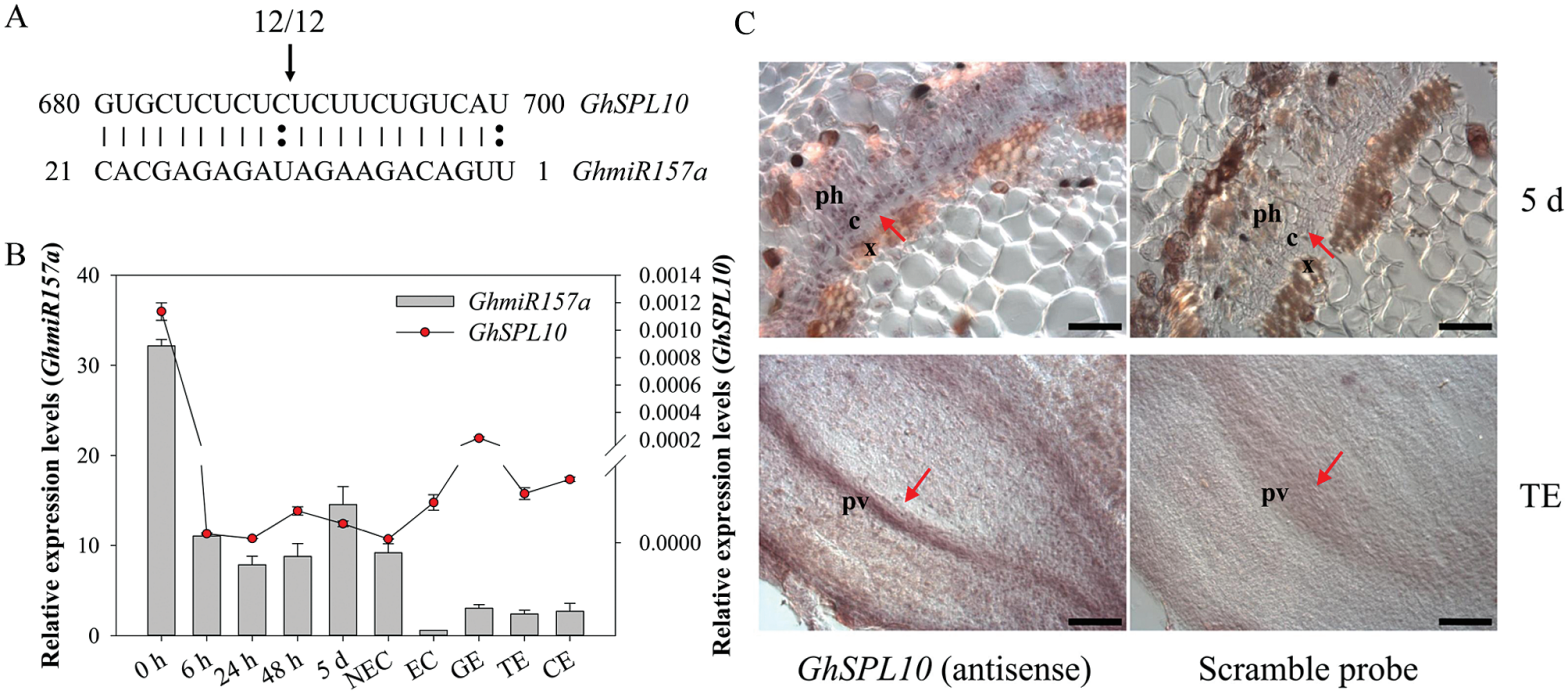
5′-RACE verification of *GhmiR157a*-guided cleavage site on *GhSPL10* mRNA and expression patterns of *GhmiR157a* and *GhSPL10* during cotton SE. (A) Cleavage site from the 5′ end for *GhSPL10* by *GhmiR157a*. The top strand depicts the mRNA fragment of *GhSPL10* complementary with *GhmiR157a*, and the bottom strand depicts *GhmiR157a* nucleotide sequence. Watson–Crick pairing (vertical dashes) and mismatches (double circles) are indicated. Cleavage site is indicated by the arrow, and the proportion of cloned 5′-RACE products corresponding to the cleavage site is indicated above the arrow. (B) qRT-PCR of *GhmiR157a* and *GhSPL10* during cotton SE. The left *y*-axis refers to the relative expression levels of *GhmiR157a* shown as grey bars, and the right *y*-axis refers to the relative expression levels of *GhSPL10* shown as red circles. Relative expression values are normalized to *UBQ7*. Values represent the mean and standard error (*n*=3). (C) Whole mount *in situ* localization of *GhSPL10* transcripts in explants cultured for 5 d (top) and torpedo-stage embryo (bottom) detected with *GhSPL10* antisense probe (left) or scramble DNA probe (right) as negative control. The red arrows indicate the cambium cells in 5 d explants (top) and provascular tissue in torpedo-stage embryo (bottom). Scale bars=50 µm. c: cambium cells; ph: phloem cells; pv: provascular tissue; x: xylem cells. (This figure is available in color at *JXB* online.)

*GhmiR156* and -*157* and their target, *GhSPL9*, were hypothesized to be involved in SE, based on previous expression data (Yang *et al.*, 2013). *AtSPL10* and *AtSPL11* are involved in zygotic embryogenesis in Arabidopsis (Nodine and Bartel, 2010), and we identified four other cotton homologues (*Gh_A12G0866*, *Gh_D12G0947*, *Gh_A11G0706*, and *Gh_D11G0821*) that exhibit strong similarity to *AtSPL10*/*11*; all harbor *GhmiR157a* target sites (Supplementary Fig. S1). We previously confirmed the cleavage sites of one homologue (*Gh_A12G0866*) by 5′-RACE (Yang *et al.*, 2013), and found that all tested clones displayed cleavage between 11 and 12 nucleotide along the *GhmiR157a* strand (Fig. 1A). This gene hereafter was referred to as *GhSPL10*.

Since *GhmiR157a* is the most abundant member of miR156 family (Yang *et al.*, 2013), we investigated the expression patterns of *GhmiR157a* and *GhSPL10* by qRT-PCR using RNA isolated from samples representing different stages of cotton SE. *GhmiR157a* and *GhSPL10* were dynamically expressed during cotton SE (Fig. 1B). Both were expressed at relatively high levels in hypocotyls, and decreased sharply during dedifferentiation and embryogenesis. Interestingly, *GhmiR157a* and *GhSPL10* exhibited inverse expression pattern in the embryonic transition stage from non-embryonic callus to embryonic callus. *GhmiR157a* showed low transcript levels during embryo formation while *GhSPL10* expression was stably low. Whole mount *in situ* hybridization was performed to determine the tissue specificity of *GhSPL10* expression; it was detected in vascular cambium of hypocotyls induced for 5 d and provascular cells of torpedo-stage embryos (Fig. 1C).

### Overexpression of *GhSPL10* promotes initial cellular dedifferentiation and callus proliferation during cotton SE

**Fig. 2.**
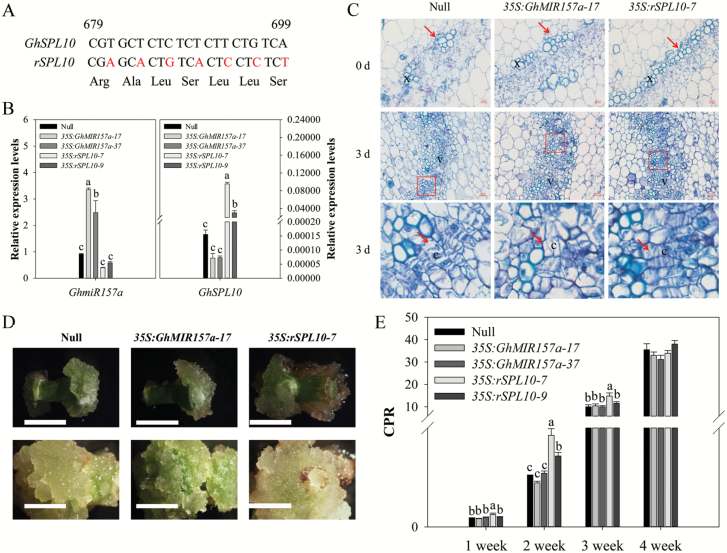
Phenotype characterizations of *GhmiR157a* and *GhSPL10* overexpression lines during cell dedifferentiation. (A) Schematic representation of *GhmiR157a*-resistant *GhSPL10* (*rSPL10*) vector constructed by synonymous mutation. The top strand depicts the original *GhmiR157a* target site of *GhSPL10* from 679 to 699 nt calculated from the start codon, and the bottom strand shows the sequence post-mutation; bases in red indicate the replaced nucleotides. (B) qRT-PCR of *GhmiR157a* and *GhSPL10* in hypocotyls of null, *GhmiR157a*, and *GhSPL10* overexpression lines. Bars represent means and standard error of three biological replicates (*n*=3). (C) Toluidine-blue stained transverse sections of explants in null, *35S:GhMIR157a-17*, and *35S:rSPL10-7* lines before culture (0 d, upper panels) and cultured for 3 d (middle and lower panels). Red arrows in the upper panels indicate xylem cells; red rectangles indicate the magnified cambium cells shown in the bottom panel indicated by the red arrows. Scale bars=20 µm for 0 d, 50 µm for 3 d. c: cambium cells; v: vascular tissue; x: xylem cells. (D) Non-embryonic callus induction phenotype in null, *35S:GhMIR157a-17*, and *35S:rSPL10-7* lines cultured for 2 (upper panels) and 4 weeks (lower panels). Scale bars=1 cm. (E) CPR in null, *GhmiR157a*, and *GhSPL10* overexpression lines cultured for 1, 2, 3, and 4 weeks. Bars represent means and standard error of five biological replicates (*n*=5). Different lower-case letters in (B, E) denote statistical differences of pairwise comparisons among each group of bars according to LSD test (*P*<0.05). (This figure is available in color at *JXB* online.)

To determine whether *GhmiR157a* and *GhSPL10* are involved in the regulation of cotton SE, a *GhmiR157a* overexpression vector was constructed as previously described (Liu *et al.*, 2017). A *GhmiR157a*-resistant *GhSPL10* overexpression vector also was constructed, with seven synonymous codon mutations at the *GhmiR157a* targeting site (Fig. 2A). Both vectors were transformed to *G. hirsutum* YZ1. In total, two *GhmiR157a* overexpression lines (*GhMIR157a-17* and *GhMIR157a-37*), two *GhSPL10* overexpression lines (*35S:rSPL10-7* and *35S:rSPL10-9*), and a null line (a negative plant line isolated from *GhSPL10* overexpression lines) were selected for further analysis (Supplementary Fig. S2A, B).

qRT-PCR was performed using RNA extracted from etiolated hypocotyls of 4-day-old seedlings, to test the expression of *GhmiR157a* and *GhSPL10* in transgenic plants (Fig. 2B). The results show that a weak reduction in the abundance of *GhSPL10* transcripts was observed in *GhmiR157a* overexpression lines, while high *GhSPL10* expression levels were observed with no alteration of *GhmiR157a* expression in *GhSPL10* overexpression lines.

The growth of hypocotyls was significantly retarded in *GhSPL10* overexpression lines, which were 1–2 cm shorter (8.38 ± 0.21 cm for *35S:rSPL10-7* and 8.99 ± 0.23 cm for *35S:rSPL10-9*) than null plants (9.99 ± 0.22 cm). In contrast, longer hypocotyls were observed in *GhmiR157a* overexpression lines, which were about 12.14 ± 0.18 cm for line 17 and 10.52 ± 0.18 cm for line 37 (Supplementary Fig. S2C, D). The concentration of endogenous IAA was measured in the different lines by HPLC-MS, and the results showed that free IAA concentration increased in the hypocotyls of *35S:rSPL10-7*, while the most prevalent IAA conjugate, IAA–Asp, decreased, implying that the IAA signaling pathway might be altered in *GhSPL10* overexpression lines. No obvious change in IAA content was observed in *35S:GhMIR157a-17* (Supplementary Fig. S2E). The production of the gaseous hormone ethylene was also found to increase significantly in *35S:rSPL10-7*, and to decrease in *35S:GhMIR157a-17*, compared with nulls (Supplementary Fig. S2E).

To determine the effects of overexpressing *GhmiR157a* and *GhSPL10* during cotton SE, calluses were induced for each transgenic line on MS medium, as described previously (Yang *et al.*, 2012). All transgenic explants showed little morphological differences in comparison with null explants cultured for 3 d. However, histological observation showed more and continuous xylem cells in the vascular system of hypocotyls in *35S:rSPL10-7* at 0 d, and even more xylem cells and more cambial cell division at 3 d, while no obvious difference was observed in vascular tissues between *35S:GhmiR157a-17* and null lines at either 0 d or 3 d (Fig. 2C).

Callus proliferation rate (CPR) was calculated among the transgenic explants during the induction period. It showed that overexpression of *GhSPL10* promoted callus proliferation at both ends of the hypocotyls during callus induction (Fig. 2D), with increasing CPR in the first 2 weeks, and thereafter a similar CPR compared with other lines (Fig. 2E). No significant change in CPR was observed in *GhmiR157a* overexpression lines compared with null at all tested time points. The CPR was higher in line *35S:rSPL10-7* compared with *35S:rSPL10-9* during the first 3 weeks post-induction, consistent with the higher expression level of *GhSPL10* in *35S:rSPL10-7* than in *35S:rSPL10-9* (Fig. 2B, E). Therefore, overexpression of *GhSPL10* accelerated callus proliferation through promoting cell division of cambium cells while *GhmiR157a* overexpression led to no obvious effects on callus formation.

### Flavonoid biosynthesis and hormonal signaling pathways induced by GhSPL10 overexpression

To explore how overexpressing *GhSPL10* regulates SE in cotton, we performed genome-wide transcript profiling of hypocotyl explants of a null line and the *35S:rSPL10-7* line. After removing adaptor contaminations and low quality tags, a total of ~12 million clean reads were generated from each library, and about 87% of the reads were mapped to cotton TM-1 genome sequences, with an ~70% gene map rate, and more than 50 000 gene transcripts were identified (Supplementary Table S2). After normalizing raw reads of each gene as fragments per kilobase of transcript per million mapped reads (FPKM), differentially expressed genes (DEGs) were identified by calculating the log2 value of the fold change comparing the FPKM of each gene in the transgenic and null line, with a threshold of 1 and probability of 0.8 of combining two biological replicates. The correlations of gene expression levels between two biological repeats were more than 0.97, indicating high uniformity between repeats (Supplementary Fig. S3A).

**Fig. 3.**
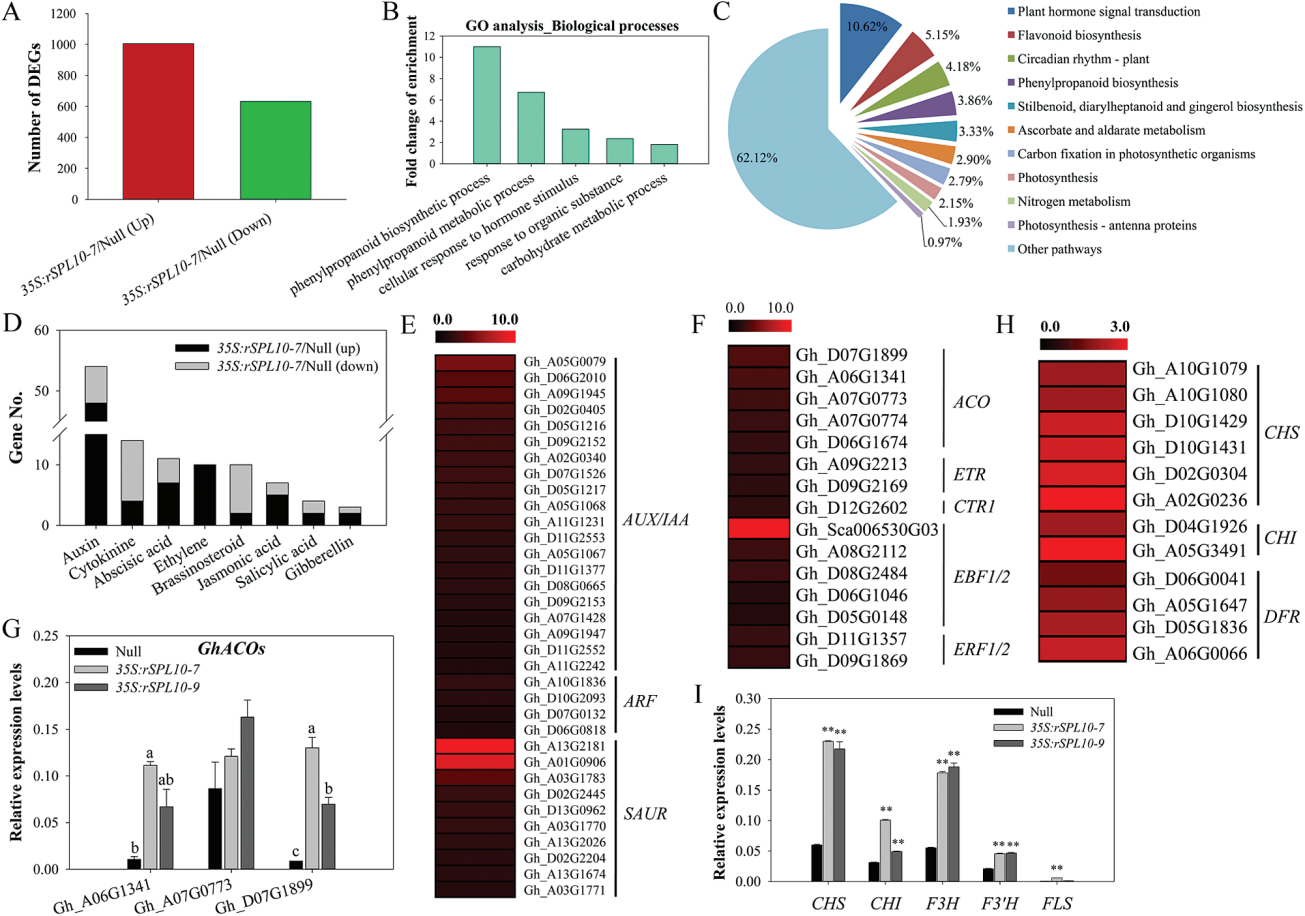
Overview of RNA sequencing data using hypocotyls of null and *35S:rSPL10-7* lines, two replicates for each line. (A) Statistics of differentially expressed gene (DEG) number between *35S:rSPL10-7* and control. The red bar represents number of up-regulated genes; the green bar represents the number of down-regulated genes. (B) GO enrichment analyses were conducted using WEGO software (Ye *et al.*, 2006), including molecular function (MF), biological process (BP), and cellular component (CC). Enrichment fold changes of genes with different BP terms were used to plot the histogram. Pearson’s chi-square test was applied to indicate significant relationships of the enrichment of each GO term, with the *P* value <0.05. (C) Proportions of DEGs among all DEGs with KEGG annotation, which include 10 significantly enriched KEGG pathways (*Q* value <0.05). (D) Number of DEGs involved in diverse hormone signaling pathways. (E) Heat map of auxin-responsive DEGs shown as log2 (*35S:rSPL10-7*/Null) values. (F) Heat map of DEGs involved in ethylene biosynthesis and signaling pathway, shown as log2 (*35S:rSPL10-7*/Null) values. (G) qRT-PCR of *ACO*s in hypocotyls of null and *GhSPL10* overexpression lines. Bars represent means and standard error of three biological replicates (*n*=3). Different lower-case letters in each group denote statistical differences of pairwise comparisons among the three lines according to LSD test (*P*<0.05). (H) Heat map of flavonoid biosynthesis-related genes shown as log2 (*35S:rSPL10-7*/Null) values. (I) qRT-PCR of flavonol biosynthesis-related genes in null and *GhSPL10* overexpression lines. Bars represented the mean and standard error of two biological replicates and three technical replicates, and statistical significance was determined by multiple comparison; **P<0.01. (This figure is available in color at *JXB* online.)

Compared with the null control, overexpression of *GhSPL10* in *35S:rSPL10-7* resulted in 1005 up-regulated genes and 633 down-regulated genes (Fig. 3A). We identified six up-regulated *SPLs*, other than *GhSPL10* (Supplementary Fig. S3B), among which *Gh_D12G1504* also had *GhmiR157a* targeting sites while the others did not (Supplementary Fig. S3C). Gene ontology (GO) analysis of differentially expressed genes revealed that genes associated with phenylpropanoid biosynthesis and cellular response to hormone stimulus were most enriched (Fig. 3B). Kyoto Encyclopedia of Genes and Genomes (KEGG) analysis further confirmed that plant hormone signaling transduction-related genes and flavonoid biosynthesis-related genes accounted for 10.62% and 5.15%, respectively, of the 932 DEGs with pathway ID (Fig. 3C). Auxin and ethylene signaling-related genes in particular were activated in *GhSPL10* overexpression lines, with the ethylene-signaling pathway genes *ETR1*, *CTR1*, *EBF1/2*, and *ERF1/2*, five *ACO* biosynthetic genes, and the auxin signal pathway *ARF* and *SAUR* genes also up-regulated in the *35S:rSPL10-7* compared with the null line (Fig. 3D–F). qRT-PCR confirmed that 2 ACO genes *Gh_A06G1341* and *Gh_D07G1899* were induced by *GhSPL10* overexpression (Fig. 3G).

**Fig. 4.**
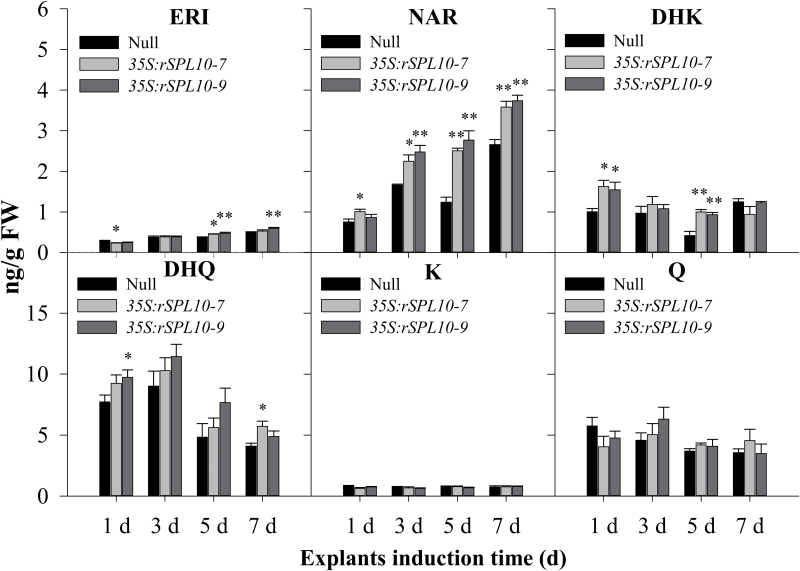
Flavonol accumulation in explants of null and *GhSPL10* overexpression lines. Quantification of ERI, NAR, DHK, DHQ, K, and Q in explants of null and *GhSPL10* overexpression lines cultured for 1, 3, 5, and 7 d. Bars are mean and standard error (*n*=4). Statistical significance was determined by multiple comparison with null as control; *P<0.05, **P<0.01.

Transcripts representing *CHS*, *CHI*, and *DFR* were activated in *GhSPL10* overexpression lines (Fig. 3H), and up-regulation of the flavonoid synthesis genes *CHS*, *CHI*, *F3H*, *F3′H*, and *FLS* was validated by qRT-PCR in *GhSPL10* overexpression lines (Fig. 3I). Flavonol assays confirmed that the contents of eriodictyol (ERI), naringenin (NAR), dihydrokaempferol (DHK), and dihydroquercetin (DHQ) were increased in *GhSPL10* overexpression lines compared with null, but no difference was observed for kaempferol (K) and quercetin (Q) (Fig. 4).

### Flavonoids promote initial cellular dedifferentiation and early cell division in cultured explants

**Fig. 5.**
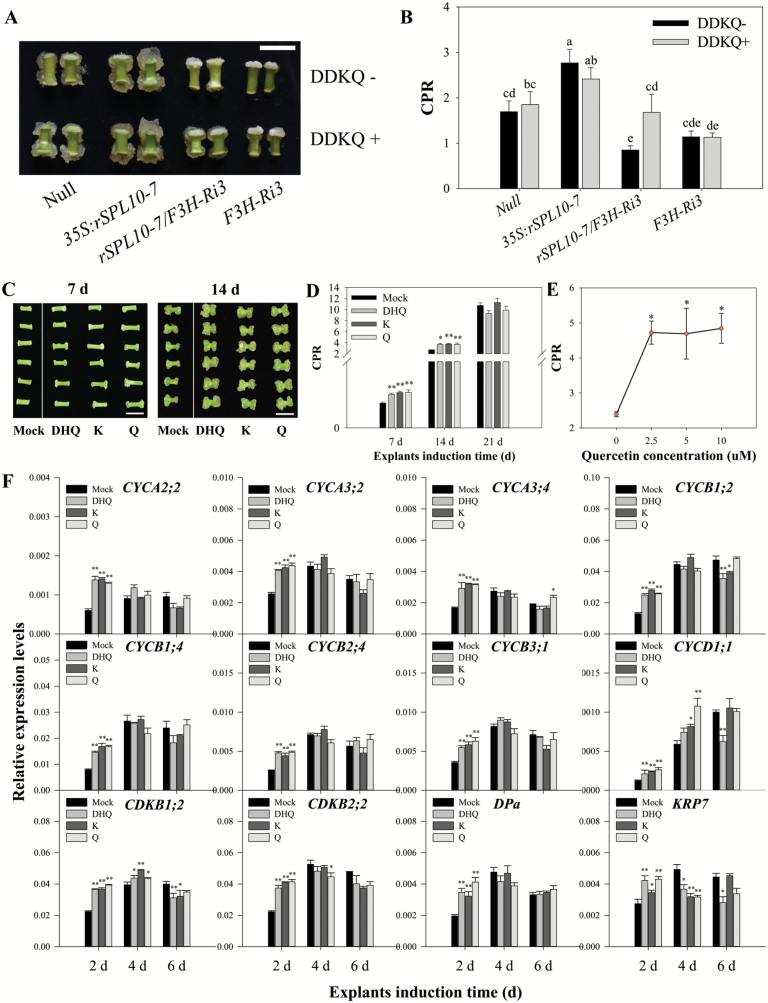
Increased flavonol accumulation in *GhSPL10* overexpression lines contributes to the higher callus proliferation rates. Callus phenotype (A) and CPR (B) of null, *35S:rSPL10-7*, *rSPL10-7*/*F3H-Ri3*, and *F3H-Ri3* lines with explants cultured on MSB medium supplemented without or with DDKQ (2.5 μM DHK, DHQ, K, and Q). Scale bar=1 cm. Bars represent means and standard error of four biological replicates (*n*=4). Different lower-case letters denote statistical differences of pairwise comparisons among all the lines and both treatments according to LSD test (*P*<0.05). (C) Callus phenotype of YZ1 explants cultured on MSB medium supplemented without (mock) or with 10 μM DHQ, K, or Q for 7 d (left) and 14 d (right). Scale bars=1 cm. (D) CPR of YZ1 explants cultured on MSB medium supplemented without (mock) or with 10 μM DHQ, K, or Q for 7, 14, and 21 d. Bars are mean and standard error (*n*=3–7). (E) CPR of YZ1 explants cultured on MSB medium supplemented with 0, 2.5, 5, and 10 μM quercetin for 2 weeks. Bars are mean and standard error (*n*=3). (F) qRT-PCR of cell cycle-related genes in YZ1 explants cultured on MSB medium supplemented without (mock) or with 10 μM DHQ, K, or Q for 2, 4, and 6 d. Bars are mean and standard error (*n*=3). Statistical significance in (D–F) was determined by multiple comparison with null as control; *P<0.05, **P<0.01. (This figure is available in color at *JXB* online.)

Since the flavonoid biosynthesis pathway was up-regulated by *GhSPL10* overexpression, we explored the link between flavonoids and the phenotypes observed in *GhSPL10* overexpression lines. To this end, we constructed a F1 generation hybrid line, *rSPL10-7*/*F3H-Ri3*, using *35S:rSPL10-7* as female parent, pollinated with pollen from an *F3H*-Ri3 RNAi line that was previously shown to accumulate reduced levels of flavonols, including DHK, DHQ, K, and Q (Tan *et al.*, 2013). The hypocotyl length of the etiolated seedlings of *rSPL10-7*/*F3H-Ri3* was 9.23 ± 0.20 cm, significantly longer than the hypocotyls of the *35S:rSPL10*-7 line (6.75 ± 0.21 cm; Supplementary Fig. S4A, B), and similar to the wild type length (9.94 ± 0.20 cm). Inhibition of F3H activity in *35S:rSPL10-7* resulted in severely reduced callus proliferation, which could be reversed to wild type levels by the *in vitro* application of DDKQ (2.5 µM DHK, DHQ, K and Q) (Fig. 5A, B). These results indicate that flavonoid overaccumulation contributes to the seedling and callus phenotypes in *GhSPL10* overexpression lines.

To test the effect of flavonols on callus induction, we treated YZ1 explants with 10 μM DHQ, K, or Q. The results show that all tested flavonols could promote callus proliferation and increase CPR at both 1 and 2 weeks (Fig. 5C, D). Further analysis using gradient concentrations of Q on MSB medium indicated that 2.5 μM Q is enough to promote callus induction, while higher levels of Q did not promote callus induction further (Fig. 5E).

The expression of cell cycle-related genes was analysed in explants cultured on MSB medium supplemented with 10 μM DHQ, K, or Q for 2, 4, and 6 days (Fig. 5F). The relative expression levels of all A, B, and D types of *CYCLIN* genes, together with *cyclin dependent kinases* (*CDKs*) and *DPa*, were activated by DHQ, K, and Q in explants cultured for 2 d, with either no effect or an inhibitory effect observed at 4 and 6 d. These results indicated that some flavonols can promote cell division during callus initiation, by up-regulating the transcription of cell cycle-related genes, and suggest that activated flavonol biosynthesis positively contributes to the enhanced callus phenotype in *GhSPL10* overexpression lines.

### Activation of ethylene signaling accelerates initial cellular dedifferentiation and mediates flavonoid biosynthesis during cotton SE

**Fig. 6.**
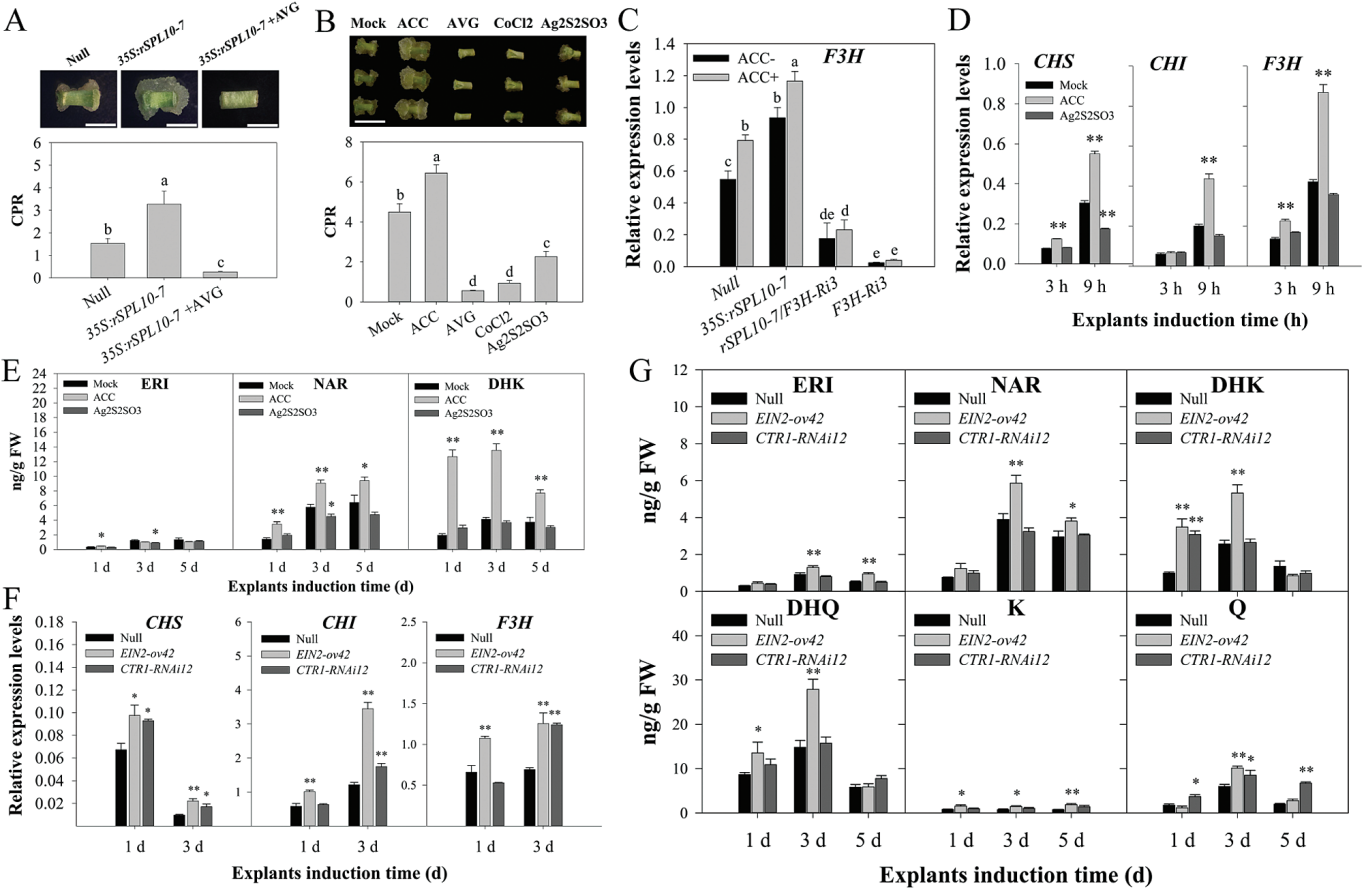
Ethylene-mediated flavonoid biosynthesis linked to callus phenotype in *35S:rSPL10-7* overexpression lines. (A) AVG treatment reversed callus phenotype (upper panel) and CPR (lower panel) in *35S:rSPL10-7*. Explants were cultured on MSB medium supplemented with or without 10 μM AVG. Scale bars=1 cm. (B) Callus phenotype (upper panel) and CPR (lower panel) for YZ1 explants treated with 10 μM ACC, 5 μM AVG, 100 μM CoCl_2_, or 10 μM Ag_2_S_2_SO_3_. Scale bar=1 cm. (C) qRT-PCR of *F3H* in explants cultured for 9 h on MSB medium supplemented with or without 25 μM ACC. (D) qRT-PCR of *CHS*, *CHI* and *F3H* in YZ1 explants cultured on MSB medium supplemented with 10 μM ACC or 10 μM Ag_2_S_2_SO_3_ for 3 and 9 h. (E) Flavonol quantification in YZ1 explants cultured on MSB medium supplemented with 10 μM ACC or 10 μM Ag_2_S_2_SO_3_ for 1, 3, and 5 d. (F) qRT-PCR of *CHS*, *CHI*, and *F3H* in explants of *EIN2-ov42* or *CTR1-RNAi12* cultured for 1 and 3 d. (G) Flavonol quantification in *EIN2-ov42* and *CTR1-RNAi12* explants cultured for 1, 3, and 5 d. Bars in (A, B, E, G) represent means and standard error of four biological replicates (*n*=4); Bars in (C, F) represent means and standard error of three biological replicates (*n*=3). Bars in (D) represent mean and standard error of two biological and three technical replicates. In (A–C) different lower-case letters on each bar graph denote statistical differences of pairwise comparisons among all bars according to LSD test (*P*<0.05). In (D–G), *P<0.05, **P<0.01. (This figure is available in color at *JXB* online.)

Since ethylene content and signaling were increased in *GhSPL10* overexpression lines, we tested whether this was related to the *GhSPL10* overexpression phenotype during SE. Inhibition of ethylene biosynthesis by AVG (an ACS inhibitor) treatment for 2 weeks during callus growth completely inhibited callus initiation in *35S:rSPL10-7*, seen as a very low CPR (Fig. 6A). This indicates that the up-regulation of ethylene biosynthesis and signaling mediates the rapid initiation of dedifferentiation and callus proliferation following *GhSPL10* overexpression.

The role of ethylene in callus proliferation was further tested in wild type explants by ACC treatment or inhibition of ethylene biosynthesis by AVG, CoCl_2_ (an ACO inhibitor), or Ag_2_S_2_SO_3_ (an inhibitor of ethylene signaling response at the receptor). It was found that ACC treatment promoted callus proliferation, while AVG, CoCl_2_, or Ag_2_S_2_SO_3_ inhibited callus initiation and proliferation (Fig. 6B).

Treatment of 9-h-cultured explants of null, *35S:rSPL10-7*, *rSPL10-7*/*F3H-Ri3*, and *F3H-Ri3* lines without or with 25 μM ACC treatment showed that ACC promotes *F3H* expression in both null and *35S:rSPL10-7* lines, but no significant enhancement of *F3H* expression levels was seen in *rSPL10-7*/*F3H-Ri3* and *F3H-Ri3* lines, presumably due to the RNAi effects on *F3H* (Fig. 6C). Expression levels of flavonoid biosynthesis-related genes were analysed by qRT-PCR in hypocotyls cultured for 3 and 9 h on MSB medium supplemented with ACC or Ag_2_S_2_SO_3_. The results show that all early flavonoid biosynthesis-related genes were up-regulated by ACC treatment, but little effect was seen when explants were treated with the ethylene response inhibitor (Fig. 6D). ACC treatment significantly increased ERI, NAR, and DHK production in callus cultured for 1, 3, and 5 d, while Ag_2_S_2_SO_3_ treatment had negligible or no significant effect on flavonol production (Fig. 6E). Enhancement of ethylene signaling using an *EIN2* overexpression line (*EIN2-ov42*) and a *CTR1* RNAi line (*CTR1-RNAi12*) (Supplementary Fig. S4C, D) led to enhanced transcription of flavonoid biosynthesis-related genes *CHS*, *CHI*, and *F3H* (Fig. 6F) and, variously, enhanced accumulation of flavonols (Fig. 6G). These results confirm the ability of the ethylene signaling pathway to activate flavonol biosynthesis.

## Discussion

### Complicated relationship of miR157-SPLs in cotton

The cleavage site of *GhSPL10* was unambiguously verified to be between nucleotides 11 and 12 in this study (Fig. 1), because the 5′-RACE cDNA samples we used here were exactly the samples reported previously (Yang *et al.*, 2013). Although the canonical cleavage site was between 10 and 11, one- or two-nucleotide shifts were also detected previously in cotton (Liu *et al.*, 2017). Besides, this phenomenon is also frequently identified through degradome sequencing, though it cannot always be detected by 5′-RACE (Yang *et al.*, 2013). Thus, multiple recognition mechanisms might exist in different tissues or developmental stages.

A positive feedback loop between miR156 and *SPL9*/*10* was reported previously (Wu *et al.*, 2009), whereby elevated expression of the miR156a precursor was seen in *AtSPL9*- and *AtSPL10*-overexpressing transgenic Arabidopsis plants. However we show in the current study that overexpression of *GhSPL10* does not alter the expression of *GhmiR157a*; on the contrary, *GhSPL10* triggers the up-regulation of one *GhmiR157a*-targeted *SPL* gene (*Gh_D12G1504*) and several non-targeted *SPLs* (Supplementary Fig. S3B, C). This phenomenon, revealed by RNA sequencing, needs further experimental validation, and more evidence is needed to illustrate the regulation mechanisms in the future.

### Flavonoids might provide antioxidant protection to modulate cell division during callus proliferation

Previous studies have shown that flavonoids can inhibit CDK activity in human cancer cells in the nanomolar concentration range and structural studies show that CDK2 can form complexes with flavonoids (De Azevedo *et al.*, 1996; Kaur *et al.*, 1992). Flavonoids extracted from various plants may be useful as human cancer therapies by promoting apoptosis and cell cycle arrest (Chen and Chen, 2013; Yasuda *et al.*, 2017). While in plants flavonoids are well known for their ecological and developmental functions, including pigmentation, defense against pathogens, signaling, and auxin transport regulation (Winkel-Shirley, 2001), their potential role in cell cycle regulation and differentiation is less obvious.

In this study, we show that overexpression of the transcription factor gene *GhSPL10* induces flavonoid biosynthesis during callus induction and cell dedifferentiation, associated with improved activity of cambium cell division. Exogenous flavonols (DHQ, K, and Q) also led to both higher CPR and higher expression levels of cell cycle-related genes compared with controls, all of which indicated a positive effect of some flavonols on cell division, which is in contrast to the roles of flavonoids in animals. However, we only tested the effect of three flavonols on cell dedifferentiation, and others may have different effects. Vigorous callus proliferation in *GhSPL10* overexpression lines is likely to be a consequence of the accumulation of one or more flavonols, and effects on cell signaling, including ethylene and auxin responses (see below). Longer exposure to high concentrations of DHQ, K, or Q may have a negative impact on cell cycle-related gene expression, consistent with the effect of such flavonols on callus induction, since higher concentrations of Q could not further promote callus induction. It is well documented that excessive accumulation of phenolics including flavonoids is correlated with tissue browning and necrosis during *in vitro* callus culture (Michael and John, 1986; Lee *et al.*, 1990; Dubravina *et al.*, 2005), which is due to the formation of quinones that inhibit callus growth in chickpea (Iqbal *et al.*, 1991), date palm (Daayf *et al.*, 2003), and cotton (Ozyigit *et al.*, 2007). Hence, flavonoids may stimulate cell division in a dose- or developmental stage-dependent manner during callus proliferation, which is associated with the modulation of cell cycle-related genes (Kosugi and Ohashi, 2003; Anzola *et al.*, 2010).

### Both auxin and ethylene signaling are modulated by *GhSPL10* during cotton SE

Multiple hormonal signaling pathways were changed by *GhSPL10* overexpression (Fig. 3), and both auxin and ethylene signaling pathways were up-regulated, implying a regulatory role for *GhSPL10* in hormonal control during cotton SE. We also found an enrichment of genes associated with circadian rhythms (Fig. 3), which themselves control multiple hormonal signaling pathways (Atamian and Harmer, 2016). It is therefore possible that photoperiod-related regulators mediate the effects of *GhSPL10* during cotton SE, but this requires further work.

### Activation of flavonoid biosynthesis by ethylene and auxin

Studies in Arabidopsis have demonstrated that SPL9 and SPL15 inhibit anthocyanin biosynthesis by competitively binding to PRODUCTION OF ANTHOCYANIN PIGMENTS1 (PAP1), which is normally bound by TRANSPARENT TESTA8 (TT8), leading to the destabilization of the MYB–BHLH–WD40 complex that activates anthocyanin biosynthesis (Gou *et al.*, 2011). These authors also found that miR156 overexpression leads to down-regulation of *CHS* and *CHI* genes, while overexpression of *MIM156* promotes their expression. It is, however, unknown whether *SPL* genes directly regulate flavonol biosynthesis-related gene expression. In this study we showed that *GhSPL10* overexpression promotes ethylene responses, and mediates flavonol biosynthesis, suggesting that *GhSPL10* might influence flavonoid biosynthesis indirectly through hormone signaling. Consistent with this, *GhSPL10* overexpression increased the conversion of auxin from the conjugated form to the free form, and several flavonoid biosynthesis-related genes were up-regulated by IAA treatment during the cell dedifferentiation stage at 2–6 d of culture (Supplementary Fig. S5). Evidence also showed that ethylene and auxin regulate flavonol biosynthesis through TIR1 and EIN2/ETR1 Lewis *et al.* (2011).

The evidence that the inhibition of ethylene biosynthesis by AVG treatment inhibited callus initiation in *GhSPL10*-overexpressing explants, and ACC treatment increased expression of flavonoid biosynthesis-related genes in wild type in very early stages of culture (3 and 9 h) (Fig. 6) further supports the importance of ethylene signaling in callus initiation. It is also possible that ethylene signaling might contribute to callus initiation and proliferation through pathways other than flavonoid biosynthesis, because inhibition of ethylene by AVG showed far more severe callus inhibition than inhibition of flavonoid biosynthesis in *35S:rSPL10-7* (Figs 5 and 6).

**Fig. 7.**
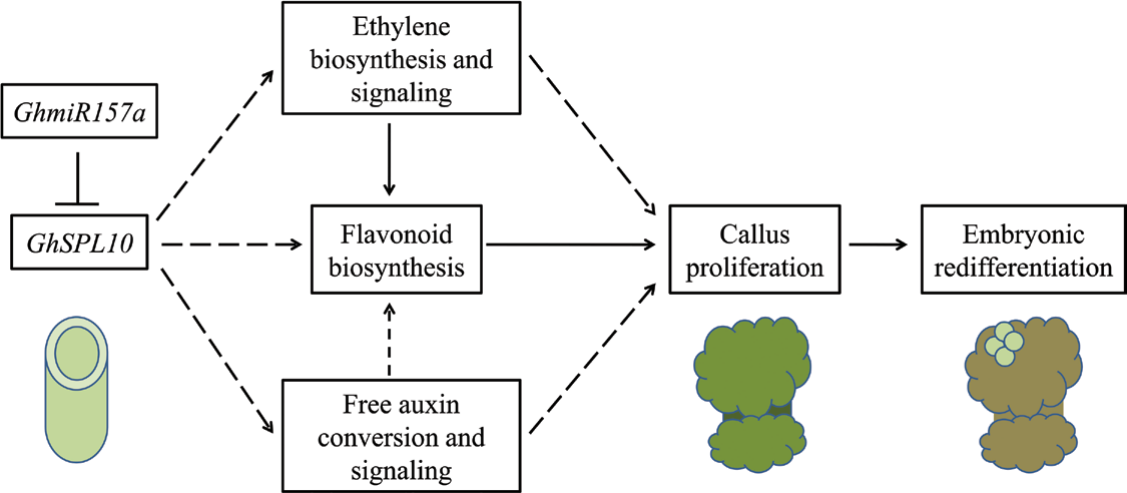
Model for the role of *GhmiR157a*–*GhSPL10* during cotton SE. During callus induction from hypocotyl explants, *GhmiR157a* negatively regulates *GhSPL10* and promotes callus induction by inducing ethylene and auxin responses, which further activate flavonoid biosynthesis to promote callus proliferation. Flavonoid accumulation in callus might also provide an antioxidant environment to facilitate embryonic fate acquisition and embryonic redifferentiation. T-shapes represent inhibition; arrows and dashed arrows indicate confirmed and probable promotion effects, respectively. The cylinder-shape represents hypocotyl explant, the dumb-bell-shapes represent explants with callus at both ends, and the circles on the callus represent regenerated globular embryos. (This figure is available in color at *JXB* online.)

In summary, we show that overexpression of *GhSPL10* activates ethylene, auxin and flavonoid pathways to regulate cell division during callus proliferation in cotton (Fig. 7).

## Supplementary data

Supplementary data are available at *JXB* online.

Fig. S1. Phylogeny of SPL proteins based on alignment of 42 SPL protein sequences in *Gossypium hirsutum* TM-1 and 15 SPLs in Arabidopsis.

Fig. S2. Molecular and seedling phenotype characterization of *GhmiR157a* and *GhSPL10* overexpression lines.

Fig. S3. Correlation analysis and identified differentially expressed *SPLs* by RNA-SEQ.

Fig. S4. Hypocotyl phenotype restoration in *35S:rSPL10-7* by inhibiting F3H activity, and transcription detection in *AtEIN2* overexpression and *CTR1* RNAi cotton plants.

Fig. S5. IAA treatment promotes flavonoid biosynthesis-related gene expression.

Table S1. List of primers used in this study.

Table S2. Summary statistics of sequencing and mapping.

Supplementary Figures S1-S5 and Tables S1-S2
