# Supplementary material for: The GhmiR157a–GhSPL10 regulatory module controls initial cellular dedifferentiation and callus proliferation in cotton by modulating ethylene-mediated flavonoid biosynthesis
Source: J Exp Bot. 2017 Dec 14;69(5):1081–93. doi: 10.1093/jxb/erx475 (PMC6018973; doi:10.1093/jxb/erx475)
Supplement: Supplementary Figures S1-S5 and Tables S1-S2 [file erx475_suppl_supplementary_figures_s1-s5_tables_s1-s2.pdf]

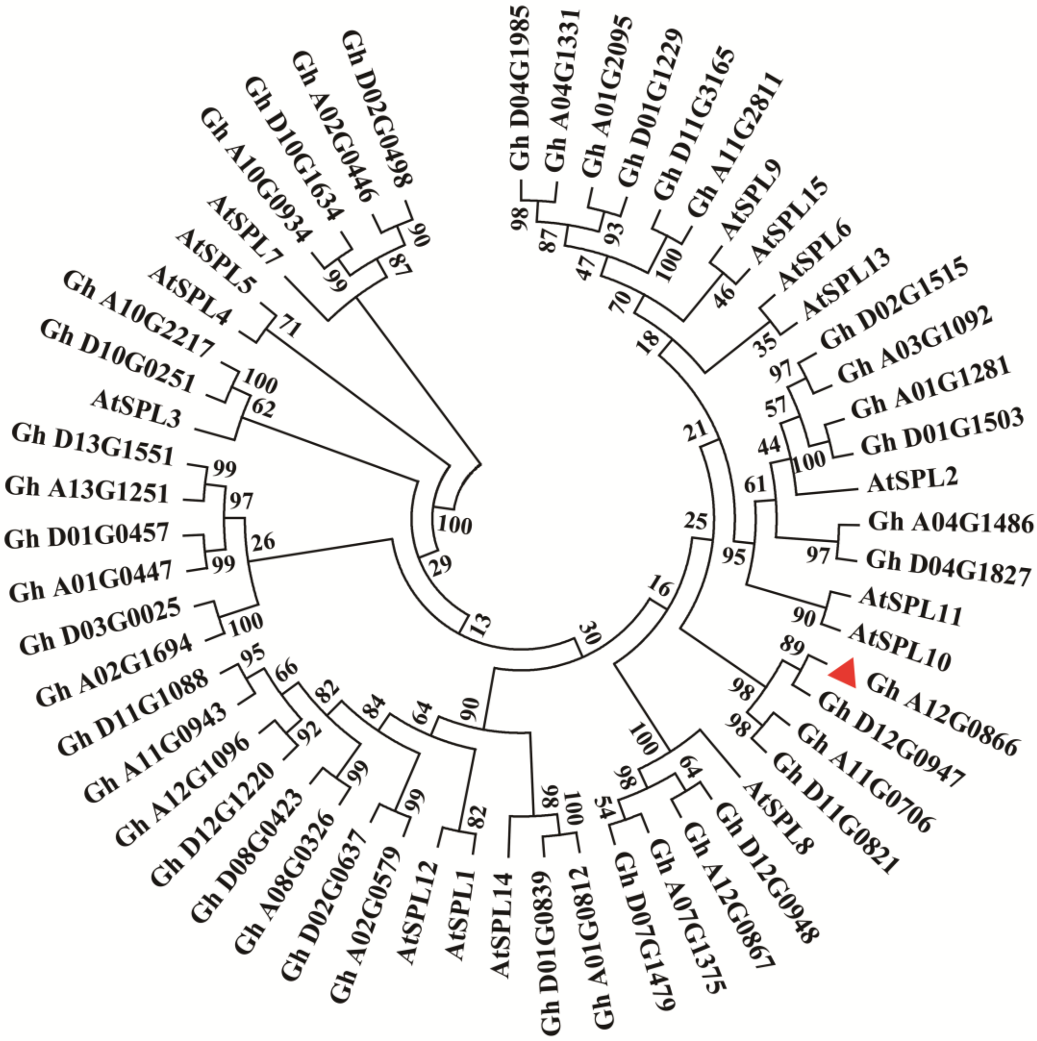


**Fig. S1.** Phylogeny of SPL proteins based on alignment of 42 SPL protein sequences in *Gossypium hirsutum* TM-1 and 15 SPLs in *Arabidopsis*. Unrooted phylogram was generated by MEGA7 software based on the neighbor-joining algorithm, with number of bootstrap replications set as 500. The red triangle indicates the *GhSPL10* focused in this study.

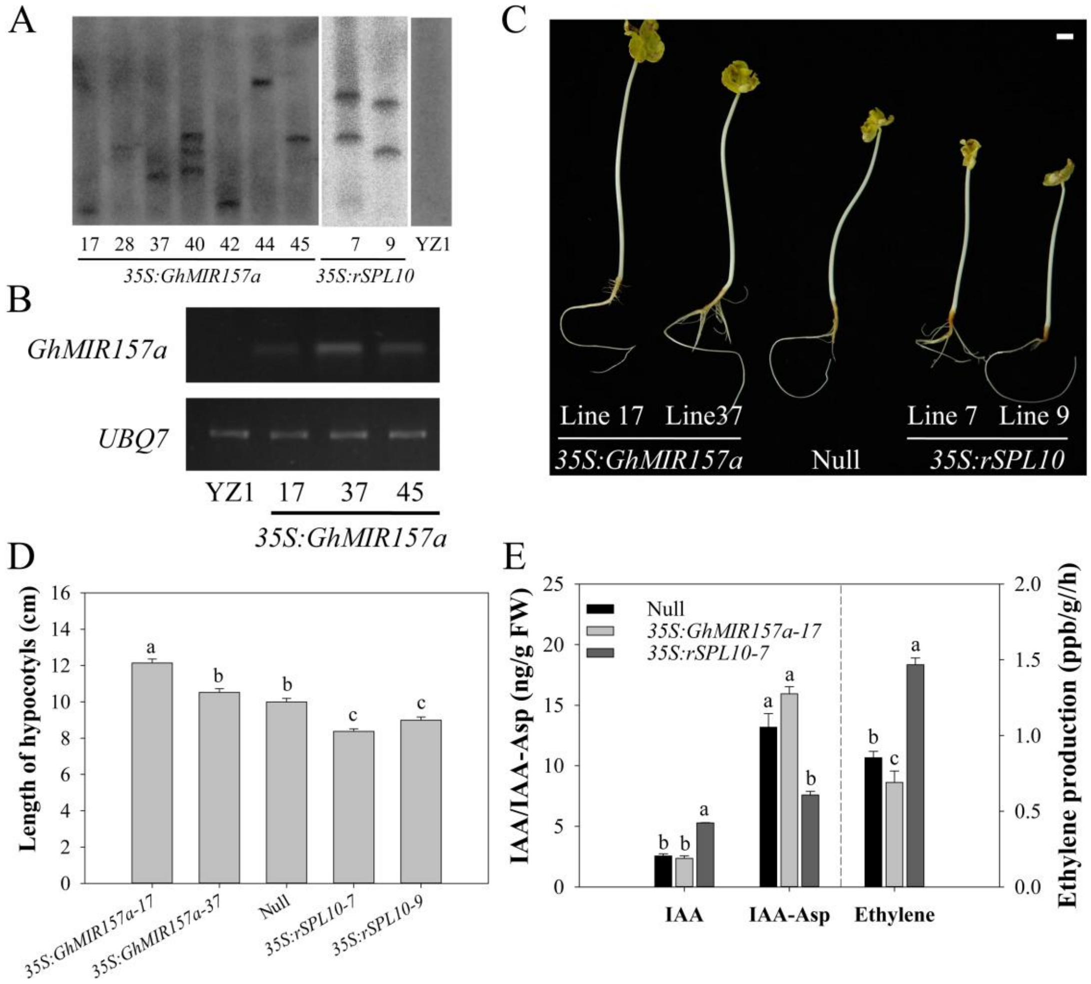


**Fig. S2.** Molecular and seedling phenotype characterization of *GhmiR157a* and *GhSPL10* overexpression lines. **(A)** Copy number identification of *GhmiR157a* and *GhSPL10* overexpression lines by Southern blotting, with YZ1 as a negative control and PCR products of *NPTII* gene as probe. **(B)** *GhmiR157a* precursor (*GhMIR157a*) detection in leaves of YZ1 and *GhmiR157a* overexpression lines by RT-PCR. *UBQ7* was used as the endogenous reference gene. **(C)** Etiolated seedling phenotype in *GhmiR157a* and *GhSPL10* overexpression lines. Scale bars = 1 cm. **(D)** Hypocotyl length of etiolated seedlings in *GhmiR157a* and *GhSPL10* overexpression lines germinated for 4 days. Error bars = mean + SE (n = 12-20). **(E)** IAA and IAA-Asp content and ethylene production in null, *35S:GhMIR157a-17* and *35S:rSPL10-7*. IAA and IAA-Asp was determined in hypocotyls of 4 d-old seedlings, and ethylene production was determined using 1 d-old germinated seeds. Error bars = mean + SE (n = 2-4). Statistical significances in **(D)** and **(E)** were determined by multiple comparison, $p < 0.05$.

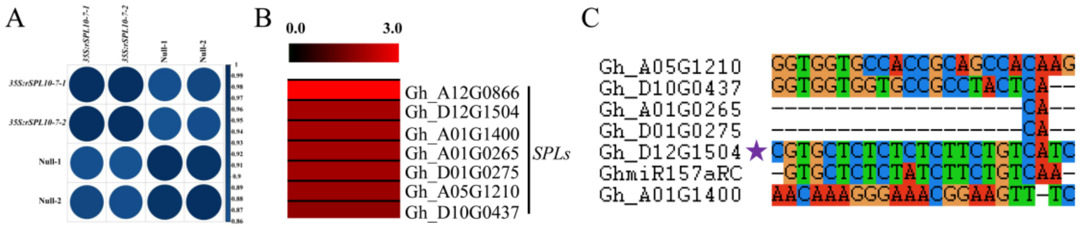


**Fig. S3.** Correlation analysis and identified differentially expressed *SPLs* by RNA-SEQ. **(A)** Correlation analysis between samples, using the Pearson method. The blue circles represent Pearson correlation coefficient (R) between each sample pair. **(B)** Differentially expressed *SPL*s between *35S:rSPL10-7* and null, shown as heat map. Scale bar displayed as gradual color change from black to red represents log2 (*35S:rSPL10-7*/Null) values from -3 to 3. **(C)** Alignment of *GhmiR157a* reverse complementary sequences (GhmiR157aRC) with differentially expressed *SPLs* identified by RNA SEQ. The purple star shows the potential *SPL* target of *GhmiR157a*. The alignment was generated by clustalX (version 1.83).

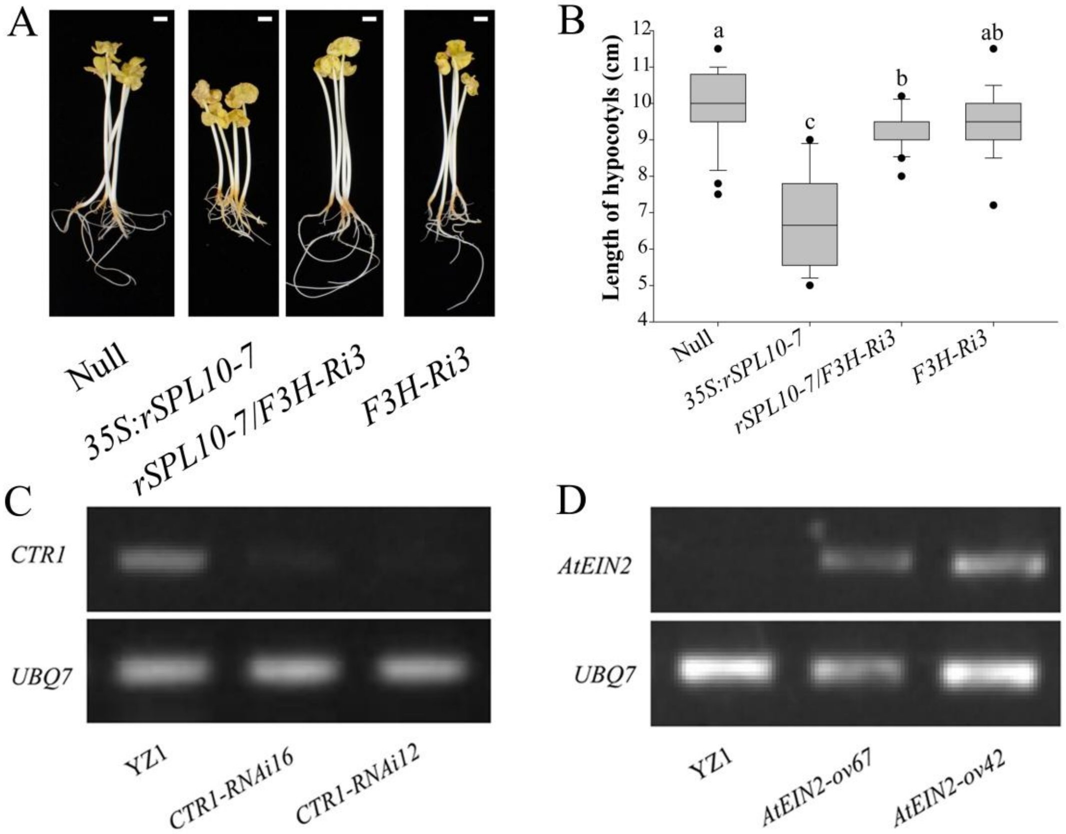


**Fig. S4.** Hypocotyl phenotype restoration in *35S:rSPL10-7* by inhibiting F3H activity, and transcription detection in *AtEIN2* overexpression and *CTR1* RNAi cotton plants. **(A)** Seedling phenotype of 5d-old etiolated seedlings of null, 35S:*rSPL10-7*, *rSPL10-7/F3H-Ri3* and *F3H-Ri3* cotton plants. Scale bars = 1 cm. **(B)** Hypocotyl length of 5d-old etiolated seedlings of null, 35S: *rSPL10-7*, *rSPL10-7/F3H-Ri3* and *F3H-Ri3* cotton plants. Error bars = mean + SE (n = 19-23). **(C)** Relative expression levels of *AtEIN2* in leaves of *EIN2-ov67* and *42* cotton plants, and **(D)** *CTR1* in *CTR1-RNAi16* and 12 cotton plants by RT-PCR, with YZ1 as control. *UBQ7* was used as an endogenous reference gene. Statistical significance in **(B)** and **(C)** was determined by multiple comparison, $p < 0.05$.

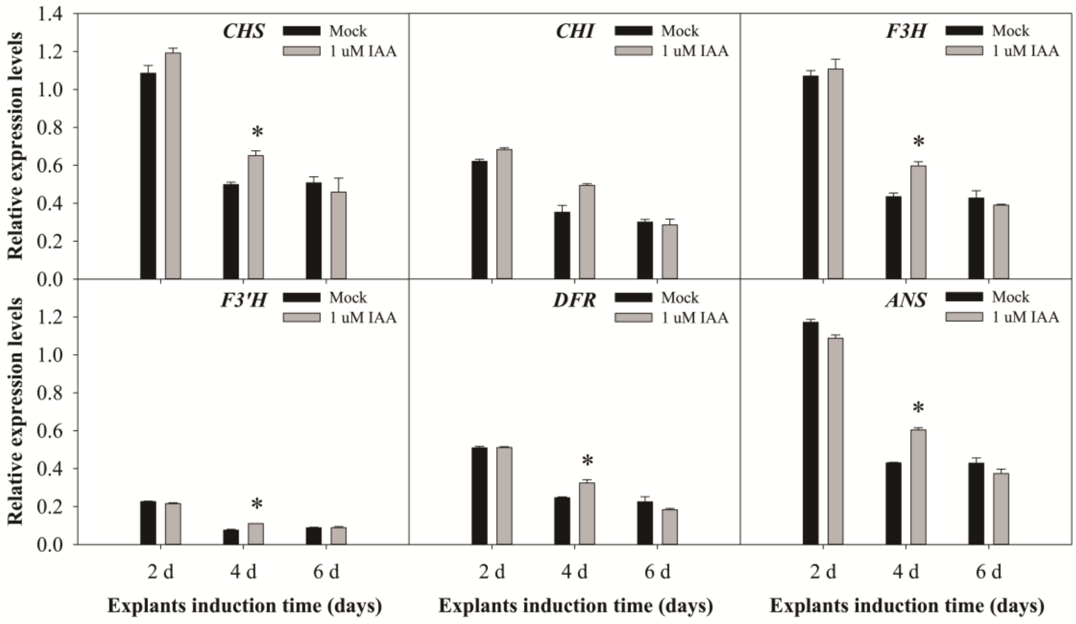


**Fig. S5.** IAA treatment promotes flavonoid biosynthesis-related gene expression. qRT-PCR of *CHS*, *CHI*, *F3H*, *F3'H*, *DFR* and *ANS* in explants of YZ1 cultured on MSB medium supplemented with 1 uM IAA for 2, 4 and 6 d respectively. Error bars represent mean and SE, statistical significance was determined by multiple comparison, *$p < 0.05$.

**Table S1.** List of primers used in this study.

| Category | Gene annotation | Gene ID | Primer | Primer Sequence |
|---|---|---|---|---|
| 5' RACE | SPL10 | Gh_A12G0866 | 3' primer | GTTCCTTGCAGGGAATGCCACAAC |
| | | | 3' nested primer | TAATGTGACTTGACCACCGATTTCC |
| In situ hybridization probe | SPL10 frag1 | Gh_A12G0866 | forward | ATGGAACATGATGATATGTTCATGGG |
| | | | reverse | TGACTAGTGTAACATTACACCCTTCAAC |
| | SPL10 frag2 | | forward | ACGCTAAAGACTATCACCGGAGAC |
| | | | reverse | CCACAATCAGCTCACGCAAGG |
| | SPL10 frag3 | | forward | AAAACCGTGCTGCTGTCCTC |
| | | | reverse | AATCACTTGGCATTTATGGTTCA |
| Overexpression | SPL10 | Gh_A12G0866 | 5' forward | GGGGACAAGTTTGTACAAAAAAGCAGGCTGAAAGAAAAAAAAGGAGAGACAA |
| | | | bridged-reverse | CCTTGGAAGAGAGGAGTGACAGTGCTCGTCCTGTTGGCGACGA |
| | | | bridged-forward | CAGGACGAGCACTGTCACTCCTCTCTTCCAAGGTTGATTCTTGGATC |
| | | | 3'reverse | GGGGACCACTTTGTACAAGAAAGCTGGGTTTCCATGTGTTCCTTGCAGGGA |
| RT-PCR | AtEIN2 | AT5G03280 | forward | AGTTGCTCCTGCGCTTTATTGT |
| | | | reverse | TTGATTCGTCTTCGTTCCTCCT |
| | CTR1 | Gh_D09G1340 | forward | CCTTAGCCAGTATCCCGACG |

| | | | | |
|---|---|---|---|---|
| | | | reverse | AAAGCCAACGCCAACTGC |
| qRT-PCR | SPL10 | Gh_A12G0866 | forward | GACTGGGAGTTTTCCGTATGTATCG |
| | | | reverse | CCACAATCAGCTCACGCAAGG |
| | ACO | Gh_D07G1899 | forward | TGGATTGGGAAAGCACATTC |
| | | | reverse | TTTGGACATGGTGGGTAGTTG |
| | | Gh_A07G0773 | forward | TACCTTCTATTTGCGCCATC |
| | | | reverse | TGCCCCATAGAAAGCCTTTT |
| | | Gh_A06G1341 | forward | GCTGGCTGAGAAACTTTTGGA |
| | | | reverse | AGGGACATCAATCCATTGGT |
| | CHS | Gh_A10G1079 | forward | TGTGTGCTCGGAGATTACTGCT |
| | | | reverse | GTTCAAACATGGGCTTCTCGAT |
| | CHI | Gh_A13G0197 | forward | TGATGCAGAAGCAAAAGCCAT |
| | | | reverse | TCCCACCTTCTGGTACTGAGCT |
| | F3H | Gh_D12G0566 | forward | GGGCCTAGCTTGCAAGCTTCTT |
| | | | reverse | AAGCAAGAGTGTGATGGTGCCTGG |
| | F3'H | Gh_D12G1798 | forward | AGTGGGAGTTGGCTGATGGATT |
| | | | reverse | CTCCTCACCCTGAAACGACAAC |
| | FLS | Gh_D05G2212 | forward | CAAGTTGTGAACCATGGCATTC |
| | | | reverse | GCCTTCAATGGATTGAGATCCT |
| | DFR | Gh_A05G1647 | forward | CGAGGACCCTGAGAATGAAGT |
| | | | reverse | GGCTTTTGTTGTTCTGCAACAT |
| | ANS | Gh_D08G1902 | forward | GAGGCCTAGCGAGCAAAATAC |
| | | | reverse | GTGAGCTTCGACACCGAGAG |
| | CYCA2;2 | Gh_D06G0428 | forward | ACTGTTGGCCTTCAGCCTAAA |
| | | | reverse | TTCTTCAACCGATGGAATCC |
| | CYCA3;2 | Gh_D06G0190 | forward | ATGGCGGGTCAGGAGAATT |

| | | | |
|---|---|---|---|
| | | reverse | GGCTGTTGTTGTTGCTGTTGT |
| CYCA3;4 | Gh_A12G0183 | forward | AGTTGATCAAGAGAACCGCGT |
| | | reverse | ACTTGGGCATTTCTCGATCT |
| CYCB1;2 | Gh_D11G3346 | forward | CCTATTGTTCCTCAACAACCC |
| | | reverse | AACTGTGCGCAAAAGGACCTA |
| CYCB1;4 | Gh_A09G1018 | forward | GCTATTGTTCCTCAGCATGAA |
| | | reverse | TGTCAACCTGCTTTCTTTGG |
| CYCB2;4 | Gh_D01G0881 | forward | TCCACACATGTGCATAGACCA |
| | | reverse | ATCCGGTCAATCTCCTCCAA |
| CYCB3;1 | Gh_A08G0724 | forward | TCAAAGAAAGGCACTGTCAGA |
| | | reverse | GCACCTTGATTTAGATGGTCC |
| CYCD1;1 | Gh_A03G1593 | forward | TCCATCAGCCGCATCTTTAT |
| | | reverse | CTCATCGGGATTGGGATAAT |
| CDKB1;2 | Gh_Sca005019G02 | forward | TACTCACTACTCCACCGCCGT |
| | | reverse | AACAGCGCGTTCCAAGTTCT |
| CDKB2;2 | Gh_D01G0840 | forward | AGGCAAATCTCAGCAATGGA |
| | | reverse | CATCAATCTGACGACATGAGG |
| Dpa | Gh_A11G2912 | forward | AATCAAACGAAACCAGCGGA |
| | | reverse | TCATGCAAACTGAATGGCGT |
| KRP7 | Gh_A03G1676 | forward | ATGGGCGATTGTATGAGAAGC |
| | | reverse | AGTCTGCAACACTTTTCACCG |
| LEC1 | Gh_D05G1686 | forward | GCCAACCATACGAACAGCCAC |
| | | reverse | GGACACACATTCTTGGATCGTTT |
| LEC2 | Gh_A09G0695 | forward | CGAAGAAGGCAACGACAACAG |
| | | reverse | GTGAACCATAAGTAGAATCCATAGGC |

| | | | |
|---|---|---|---|
| GhUBQ7 | Gh_A11G0969 | forward | GAAGGCATTCCACCTGACCAAC |
| | | reverse | CTTGACCTTCTTCTTCTTGTGCTTG |

**Table S2.** Summary statistics of sequencing and mapping.

| Sample Name | Clean reads | Genome map Rate | Gene map Rate | Expressed Gene |
|---|---|---|---|---|
| Null-1 | 12154146 | 86.87% | 69.37% | 50359 |
| Null-2 | 12162581 | 86.60% | 69.84% | 51011 |
| *35S:rSPL10-7-1* | 12163262 | 86.74% | 71.34% | 50911 |
| *35S:rSPL10-7-2* | 12160933 | 86.56% | 71.83% | 51216 |
